# Exposures to Elevated Core Temperatures during Football Training: The Impact on Autonomic Nervous System Recovery and Function

**DOI:** 10.3390/sports12010008

**Published:** 2023-12-27

**Authors:** Eric Renaghan, Harrison L. Wittels, Luis A. Feigenbaum, Michael J. Wishon, Stephanie Chong, Eva D. Wittels, Stephanie Hendricks, Dustin Hecocks, Kyle Bellamy, Joe Girardi, Stephen Lee, Tri Vo, Samantha M. McDonald, S. Howard Wittels

**Affiliations:** 1 Department of Athletics, Sports Science, University of Miami, Miami, FL 33146, USA; eric.renaghan@miami.edu (E.R.); lfeigenbaum@med.miami.edu (L.A.F.); 2 Tiger Tech Solutions, Inc., Miami, FL 33156, USA; hl@tigertech.solutions (H.L.W.); joe@tigertech.solutions (M.J.W.); schong591@gmail.com (S.C.); evadanielle@gmail.com (E.D.W.); steph.hendricks@gmail.com (S.H.); dustin@tigertech.solutions (D.H.); shwittels@gmail.com (S.H.W.); 3 Department of Physical Therapy, Miller School of Medicine, University of Miami, Miami, FL 33146, USA; j.girardi@miami.edu; 4 Department of Athletics, Nutrition, University of Miami, Miami, FL 33146, USA; k.bellamy1@umiami.edu; 5 United States Army Research Laboratory, Adelphi, MD 20783, USA; stephen.j.lee28.civ@mail.mil; 6 Navy Medical Center—San Diego, San Diego, CA 92134, USA; huu@g.clemson.edu; 7 School of Kinesiology and Recreation, Illinois State University, Normal, IL 61761, USA; 8 Department of Anesthesiology, Mount Sinai Medical Center, Miami, FL 33140, USA; 9 Department of Anesthesiology, Wertheim School of Medicine, Florida International University, Miami, FL 33199, USA; 10 Miami Beach Anesthesiology Associates, Miami, FL 33140, USA

**Keywords:** temperature, collegiate, American football, autonomic nervous system, recovery, exercise intensity, strength and conditioning, exercise duration

## Abstract

Exercising with elevated core temperatures may negatively affect autonomic nervous system (ANS) function. Additionally, longer training duration under higher core temperatures may augment these negative effects. This study evaluated the relationship between exercise training duration and 24 h ANS recovery and function at ≥37 °C, ≥38 °C and ≥39 °C core temperature thresholds in a sample of male Division I (D1) collegiate American football athletes. Fifty athletes were followed over their 25-week season. Using armband monitors (Warfighter Monitor^TM^, Tiger Tech Solutions, Inc., Miami, FL, USA), core temperature (°C) and 24 h post-exercise baseline heart rate (HR), HR recovery and heart rate variability (HRV) were measured. For HRV, two time-domain indices were measured: the root mean square of the standard deviation of the NN interval (rMSSD) and the standard deviation of the NN interval (SDNN)**.** Linear regression models were performed to evaluate the associations between exercise training duration and ANS recovery (baseline HR and HRV) and function (HR recovery) at ≥37 °C, ≥38 °C and ≥39 °C core temperature thresholds. On average, the athletes were 21.3 (± 1.4) years old, weighed 103.0 (±20.2) kg and had a body fat percentage of 15.4% (±7.8%, 3.0% to 36.0%). The duration of training sessions was, on average, 161.1 (±40.6) min and they ranged from 90.1 to 339.6 min. Statistically significant associations between training duration and 24 h ANS recovery and function were observed at both the ≥38.0 °C (baseline HR: *β* = 0.10 ± 0.02, R^2^ = 0.26, *p* < 0.0000; HR recovery: *β* = −0.06 ± 0.02, R^2^ = 0.21, *p* = 0.0002; rMSSD: *β* = −0.11 ± 0.02, R^2^ = 0.24, *p* < 0.0000; and SDNN: *β* = −0.16 ± 0.04, R^2^ = 0.22, *p* < 0.0000) and ≥39.0 °C thresholds (*β* = 0.39 ± 0.05, R^2^ = 0.62, *p* < 0.0000; HR recovery: *β* = −0.26 ± 0.04, R^2^ = 0.52, *p* < 0.0000; rMSSD: *β* = −0.37 ± 0.05, R^2^ = 0.58, *p* < 0.0000; and SDNN: *β* = −0.67 ± 0.09, R^2^ = 0.59, *p* < 0.0000). With increasing core temperatures, increases in slope steepness and strengths of the associations were observed, indicating accelerated ANS deterioration. These findings demonstrate that exercise training under elevated core temperatures (≥38 °C) may negatively influence ANS recovery and function 24 h post exercise and progressively worsen.

## 1. Introduction

Achieving peak sport performance relies on optimal functioning of the autonomic nervous system (ANS) [1,2]. The ANS plays an integral role in thermoregulation, a process that provides homeostatic control of core body temperatures, typically between 36.1 °C and 37.8 °C [3]. During exercise, core temperature increases as skeletal muscle metabolism quickly accelerates heat production [4]. Slight elevations in core temperature improve energy efficiency and performance due to increased enzymatic activity and O_2_ diffusion into skeletal muscle [4]. As core temperature rises, the ANS thermoregulates via evaporation, the primary method for controlling internal temperature during exercise [4]. To dissipate heat, the peripheral vasculature vasodilates, shunting the blood away from the working skeletal muscles to the skin, activating the sweat glands [4]. Subsequently, the body starts to sweat, inducing heat loss [4]. However, any perturbations inhibiting evaporation like high external heat, dehydration, high humidity and restrictive clothing may further increase core temperature [5,6,7]. A persisting increase in core temperature indicates the inability of the ANS to adequately thermoregulate, potentially leading to ANS deterioration.

Several studies previously evaluated the negative effects of high heat exposure during exercise training among athletes [8,9,10]. Collectively, these studies demonstrated that athletes, when training in high heat, may experience reductions in cardiac output, increased carbohydrate metabolism, glycogen depletion, dehydration, an accelerated decline in performance and increased risk of exertional heat illnesses (EHIs) like heat exhaustion (38.5 °C–39.9 °C) and/or heat stroke (≥40.0 °C) [8,9,10]. While these *acute effects* are significant, it is possible that disturbances to ANS recovery and function occur prior to these observable effects and persist the following day. Importantly, to our knowledge, no studies have previously evaluated the potential longer-term influence of training in high heat on 24 h ANS recovery and function. Additionally, while duration of training undoubtedly influences the severity of effects consequent to training in high heat, former studies did not evaluate the relationship between training duration and 24 h ANS recovery and function under specific core temperature thresholds. As such, there is a significant gap in knowledge on whether ANS dysfunction persists beyond the immediate post-training period and if prolonged training duration at sub-hyperthermic and hyperthermic levels influences ANS recovery and function. Lastly, a vast majority of previous studies were conducted for endurance-based sports, like long-distance cycling and marathon running; thus, much less is known for non-endurance-based sports like American collegiate football [11,12].

Understanding the potential longer-term effects of heat exposure among American collegiate football athletes is an imminent concern because these athletes are at a higher risk of heat injury [13,14,15]. The increased risk is due to several factors including prolonged, consecutive training sessions (e.g., 2 to 4 h), the inclusion of high-intensity activities and wearing heat-intolerant clothing/equipment [6,16,17]. Knowing the functional limits of the ANS to heat exposure may allow coaches to structure training programs more effectively in hot and humid climates (≥32.2 °C and ≥30% relative humidity) to prevent ANS dysfunction and optimize sports performance. Therefore, the purpose of this study was to evaluate the influence of varying core temperatures on the relationship between training duration and 24 h ANS function throughout a 25-week collegiate football season. We hypothesized that prolonged training sessions under sub-hyperthermic and hyperthermic core temperatures would negatively influence 24 h ANS recovery and function.

## 2. Materials and Methods

### 2.1. Study Design

The current study employed a prospective cohort design that tracked ANS recovery and function 24 h post-exercise training sessions over a 25-week season in a sample of male Division I collegiate American football athletes. Indicators of ANS recovery and function were baseline heart rate (HR), HR recovery and heart rate variability (HRV).

### 2.2. Subjects

Male subjects were recruited from the current year’s roster of a D1 collegiate American football team located in southeast Florida, United States. Fifty healthy, male football athletes participated in this study and, on average, were 21.3 (±1.4) years old, weighed 103.0 (±20.2) kg, were 187.5 (±6.6) cm tall, 70.0% non-Hispanic black, 20.0% Caucasian, and 10.0% Hispanic. The anthropometric profiles of the athletes varied widely with body mass indices and percent body fat ranging from 23.7 kg/m^2^ to 44.9 kg/m^2^ and 3.0% to 36.0%, respectively. Prior to study participation, the athletes were informed of the benefits and risks of the study and voluntarily consented to the study. Informed consent was obtained from all subjects involved in the study. All study protocols followed the ethical principles defined in the Declaration of Helsinki and were approved by the university’s Institutional Review Board (IRB #20191223).

### 2.3. Procedures

#### 2.3.1. Exercise Training Sessions

**Figure 1:**
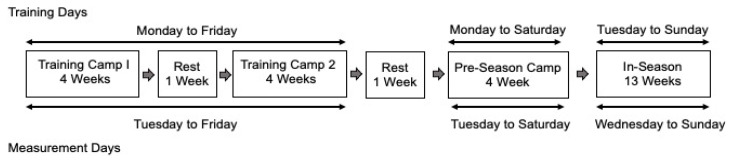
Prospective study design for exercise training sessions and measurement days.

Fifty collegiate American football athletes participated during their 27-week season consisting of two 4-week summer camps, each separated by a week of rest, one 4-week pre-season camp and a 13-week in-season (see Figure 1). The exercise training sessions lasted, on average, 161.1 (±40.6) min and ranged from 90.1 to 339.6 min. Although the intensity and exercises performed varied within and between training sessions, all athletes were exposed to the same training. All training occurred during football practices and incorporated strength- and power-focused resistive exercises, short-distance sprint intervals, aerobic training and agility training.

#### 2.3.2. Measurements

Participants wore armband monitors equipped with temperature and electrocardiography (ECG) capabilities (Warfighter Monitor (WFM), Tiger Tech Solutions Inc., Miami, FL, USA). The WFM was previously validated in many subpopulations, including athletes [18,19,20,21]. Monitors were secured with an elastic band placed on the upper left arm and worn throughout exercise training sessions. Core temperature, HR, HRV and duration were all measured using the WFM and are described in further detail below.

#### 2.3.3. Core Temperature

Core body temperature was derived from temperature sensors on the WFM. The temperature sensors on the WFM are medical-grade infrared temperature sensors which have been calibrated to core body temperature across thousands of subjects in a hospital setting utilizing health monitors and Swan–Ganz catheters as the gold standard measure [22,23]. Core temperature was measured throughout the duration of each training session.

#### 2.3.4. 24 h ANS Recovery and Function

The current study used cardiac-based metrics to represent ANS recovery and function given that cardiac activity is a strong indicator of ANS activity. The release of acetylcholine and epinephrine from cardiac nerve fibers controls the firing rate of the sinoatrial (SA) node affecting cardiac activity. Thus, cardiac metrics like baseline HR, HR recovery and heart rate variability (HRV) provide accurate information on the interaction between parasympathetic and sympathetic nervous systems, the two branches of the ANS.

#### 2.3.5. Baseline HR

Baseline HR represented ANS recovery and was measured in the morning (0600–0700), prior to the start of a football training session and 24 h after the start of the previous day’s training session. Baseline HR was obtained following at least 4 min of inactivity, per established protocols [24]. During this measurement, athletes remained seated and nearly motionless for 5 min to collect a “resting” baseline HR.

#### 2.3.6. HR Recovery

HR recovery was measured throughout the next day’s football training session, 24 h following the previous day’s training session. HR recovery was defined as the reduction in HR during 30 s rest intervals and represented localized parasympathetic activation. HR recovery was measured within the first 30 s of the rest interval, as during this period HR exhibits the greatest rate of change following an acute bout of exercise [25]. HR recovery was estimated during all rest intervals occurring within a training session and then averaged.

#### 2.3.7. HRV

Like baseline HR, HRV was measured in the morning (0600–0700), prior to the start of a football training session and 24 h after the start of the previous day’s training session. Per established protocols [24], HRV was obtained following at least 4 min of inactivity. Two HRV time-domain indices were measured which assessed the changes in the inter-beat interval including RR and NN intervals. RR intervals were the time between R waves on consecutive QRS complexes and NN intervals were noise-free RR intervals. From these data, the two separate time-domain indices were derived including SDNN (standard deviation of the NN interval) and RMSSD (the root mean square of the standard deviation of the NN interval). These HRV time-domain indices have been shown to reflect parasympathetic autonomic output [26].

Because the current study focused on ANS recovery and function 24 h post-exercise training, baseline HR, HR recovery and HRV were not measured following one or more rest days. For example, baseline HR, HR recovery and HRV were not measured on Mondays during summer training camps as this day was preceded by two rest days (Saturday and Sunday). The inclusion of rest days in the analyses would likely weaken the associations and, thus, less accurately estimate the acute impact of exercise training duration and core temperature on short-term ANS recovery and function (see Figure 1).

### 2.4. Statistical Analysis

The current study evaluated the relationships between acute exercise training duration, varying core temperature thresholds and the influence on ANS recovery and function within 24 h post exercise. The primary independent variables were exercise training duration (in minutes) and core temperature (°C). The measures of ANS recovery and function including 24 h HR, HR recovery and HRV, specifically SDNN and rMSSD, served as the primary outcome variables. The relationships between exercise training duration and ANS recovery and function were stratified by data-driven core temperature thresholds: ≥37 °C, ≥38 °C and ≥39 °C. All conditional associations exhibited a normal distribution, evaluated via the Kolmogorov–Smirnov test. The relationships of interest were estimated using linear regression models and were performed separately for each core temperature threshold and outcome variable. For all models, β coefficients and standard errors were estimated, and the a priori threshold for statistical significance was set at α = 0.05. Statistical analyses were performed in MATLAB, version 2021b (MathWorks, Natick, MA, USA).

## 3. Results

The duration of training sessions under specific temperature thresholds and the athletes’ 24 h ANS recovery and function are presented in Table 1. The average duration of training sessions was 161.1 (±40.6) min. The average time athletes spent in each temperature threshold decreased with increasing temperature from 30.4 (±35.9) min at a core temperature ≥ 37 °C to 10.5 (±12.9) min at a core temperature ≥ 39 °C. For 24 h ANS recovery, the athletes, on average, exhibited a baseline HR of 61.4 (±8.6) bpm, ranging between 44.8 and 118.2 bpm. Following acute bouts of exercise performed 24 h post training, the athletes exhibited HR recovery values of 30.6 (±6.0) bpm, ranging between 11.2 and 49.6 bpm, during 30 s rest intervals. For HRV post 24 h, athletes exhibited, on average, rMSSD values of 72.0 ms (SD ± 7.0, range: 55.4 to 93.2) and SDNN values of 106.7 ms (SD ± 107.6, range: 81.1 to 141.8).

**Figure 2:**
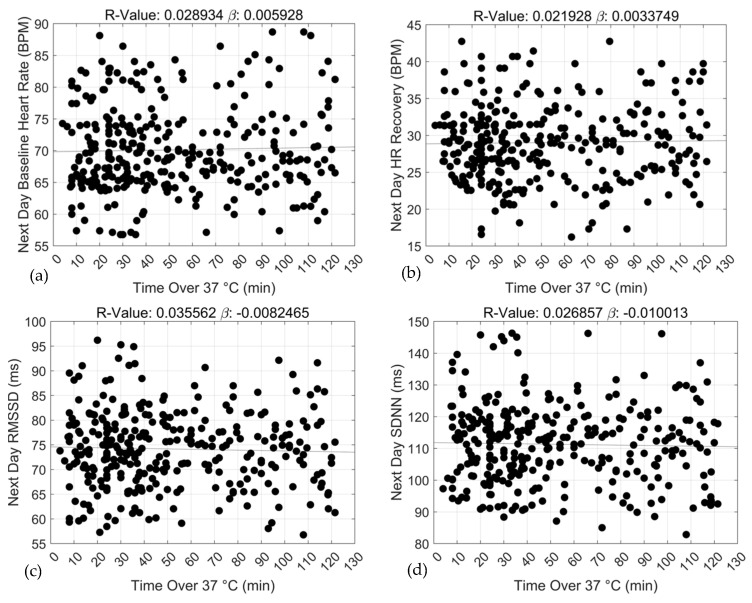
Associations between exercise training duration (min) and 24 h baseline heart rate (**a**), 24 h heart rate recovery (**b**), 24 h rMSSD (**c**) and 24 h SDNN (**d**) over the ≥37 °C threshold.

**Figure 3:**
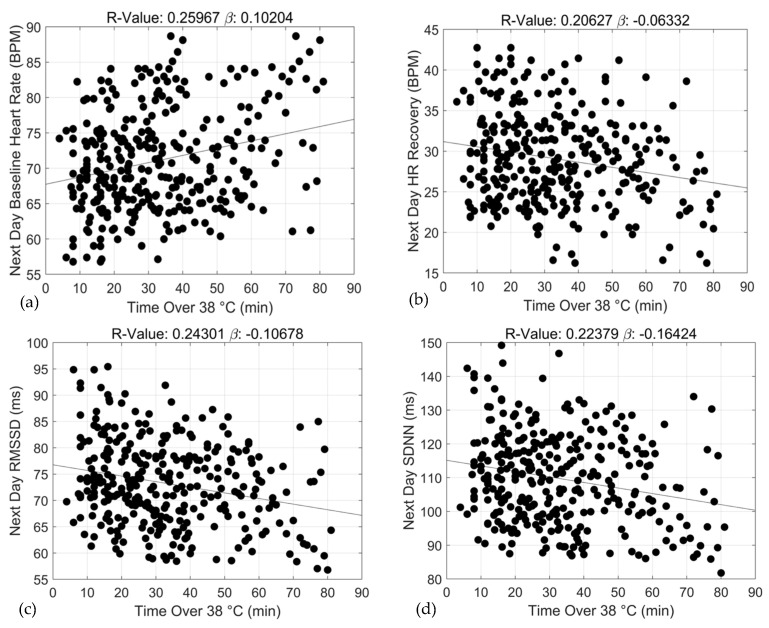
Associations between exercise training duration (min) and 24 h baseline heart rate (**a**), 24 h heart rate recovery (**b**), 24 h rMSSD (**c**) and 24 h SDNN (**d**) over the ≥38 °C threshold.

**Figure 4:**
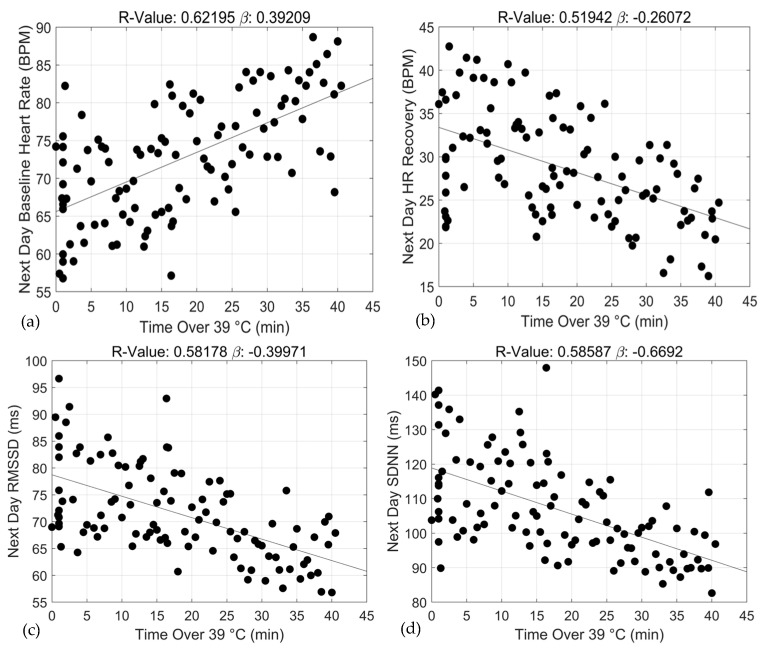
Associations between exercise training duration (min) and 24 h baseline heart rate (**a**), 24 h heart rate recovery (**b**), 24 h rMSSD (**c**) and 24 h SDNN (**d**) over the ≥39 °C threshold.

Adjusted linear regression and correlation coefficients estimating the relationships between exercise training duration under specific core temperature thresholds and 24 h ANS recovery and function are shown in Table 2. For baseline HR, statistically significant associations with 24 h recovery were observed for two of the three core temperature thresholds including ≥38.0 °C (*β* = 0.10 ± 0.02, R^2^ = 0.26, *p* < 0.0000) and ≥39.0 °C (*β* = 0.39 ± 0.05, R^2^ = 0.62, *p* < 0.0000). Similar associations were observed for 24 h ANS function including ≥38.0 °C (*β* = −0.06 ± 0.02, R^2^ = 0.21, *p* < 0.0002) and 39.0 °C (*β* = −0.26 ± 0.04, R^2^ = 0.52, *p* < 0.0000). At higher core temperature thresholds, specifically ≥38.0 °C and 39.0 °C, duration of training sessions was positively and negatively associated with 24 h baseline HR and HR recovery, respectively. Moreover, the strengths of the associations between duration and ANS recovery and function increased with increases in core temperature, ≥38.0 °C and ≥39.0 °C bpm (baseline HR: *β* range = 0.10 vs. 0.39, R^2^: 0.26 vs. 0.62; and HR recovery: *β* range = −0.06 vs. −0.26, R^2^ = 0.21 vs. 0.52). At the lowest core temperature threshold (≥37.0 °C), duration of training was not significantly associated with ANS recovery and function. Graphical representations of these relationships appear in Figure 2, Figure 3 and Figure 4.

Table 3 presents the adjusted linear regression and correlation coefficients for the associations between time spent training under specific core temperature thresholds and HRV indices. For rMSSD, statistically significant associations with training duration associations were observed at the ≥38 °C and ≥39 °C thresholds (*β* = −0.11 ± 0.02, R^2^ = 0.24, *p* < 0.0000; and *β* = −0.37 ± 0.05, R^2^ = 0.58, *p* < 0.0000). Under the ≥37 °C threshold, a weak, non-statistically significant association between training duration and rMSSD was observed. Like rMSSD, statistically significant associations between training duration and SDNN were found at the ≥38 °C and ≥39 °C thresholds (*β* = −0.16 ± 0.04, R^2^ = 0.22, *p* < 0.0000; and *β* = −0.67 ± 0.09, R^2^ = 0.59, *p* < 0.0000). At ≥37 °C, however, the weak association between training duration and SDNN did not reach statistical significance. Similar to 24 h baseline HR and HR recovery, the strengths of the associations for rMSSD and SDNN increased with an increase in the temperature threshold, whereby a higher core temperature resulted in a greater decline in HRV when exercising for longer durations.

## 4. Discussion

The purpose of this study was to evaluate the influence of varying core temperature thresholds on the relationship between training duration and 24 h ANS function throughout a 25-week collegiate football season. We hypothesized that prolonged training sessions under sub-hyperthermic and hyperthermic core temperatures would negatively influence 24 h ANS recovery and function. The major findings of this study were that (1) athletes training longer at temperatures ≥38.0 °C and ≥39.0 °C negatively influenced ANS recovery and function 24 h later, (2) the strengths of these associations increased with increases in core temperature, (3) the training duration that led to a similar magnitude of ANS deterioration differed between the ≥38.0 °C and ≥39.0 °C thresholds and (4) when including core temperatures between 37.0 °C and 37.9 °C, statistical significance no longer held between duration of training and 24 h ANS recovery and function.

The most novel aspect of this study was observing reduced ANS recovery and function 24 h following exercise training at core temperature thresholds ≥38.0 °C and ≥39.0 °C. At these thresholds, training duration was associated with a progressively negative impact on 24 h baseline HR, HR recovery and HRV. That is, athletes training longer at core temperatures ≥38.0 °C and ≥39.0 °C, on average, showed signs of ANS deterioration. Specifically, the observed higher baseline HR, slower HR recovery and depressed HRV suggest that athletes had residual elevated sympathetic activity and reduced parasympathetic outflow 24 h post training. This finding significantly expands upon the existing evidence and highlights two important concepts. First, our study showed that training at higher core temperatures may elicit a longer-lasting negative impact on the ANS than previously demonstrated. Former studies primarily focused on ANS recovery in the immediate post-training period, typically following a competition [11,12]. After competitions, most athletes refrain from moderate- and high-intensity exercise for at least 48 h before returning to training [27,28]. As such, evaluating ANS function 24 h post competition was possibly not relevant and its significance remained unclear. Second, previous research largely evaluated, understandably, the acute effects of elevated core temperatures, such as decrements in performance, dehydration, cognitive deficits, etc. [10,29]. Of concern is that this potentially assumes that signs of heat-related ANS dysfunction are only present if observable. However, it is possible that not all athletes exhibit noticeable symptoms of ANS degradation and, consequently, it can remain undetected and may persist the following day, as demonstrated in our study. More importantly, collegiate American football often requires athletes to train 5 to 6 days per week, meaning that training sessions often occur on consecutive days. This could be problematic for athletes training in hot and humid climates if ANS dysfunction consequent to higher core temperatures remains undetected and athletes continue practicing. These cumulative exposures to training with ANS dysfunction and/or elevated core temperature may further perpetuate ANS deterioration, suboptimal performance and greatly increase the risk of exertional heat illness. Thus, for optimizing sports performance, this study finding suggests coaches should frequently monitor core temperature and duration of exposure, especially during summer training camps and preseason [16,30].

**Figure 5:**
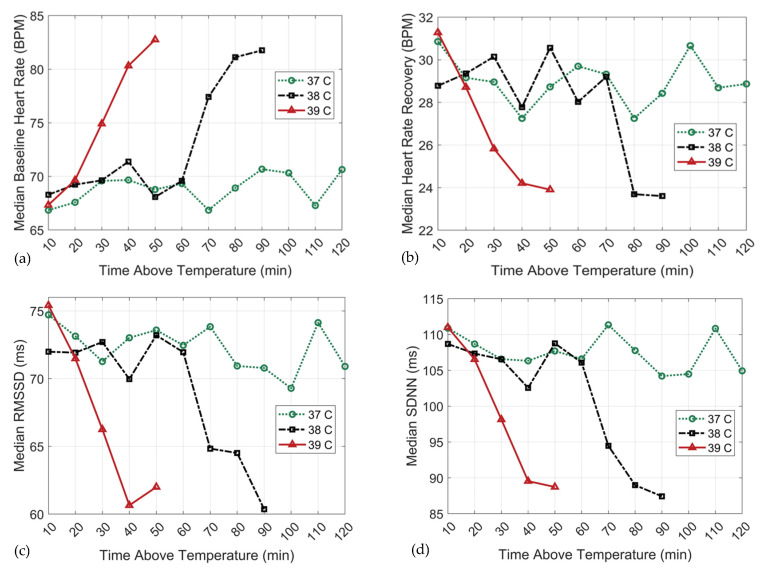
Comparison of the associations between exercise training duration (min) and 24 h baseline heart rate (**a**), 24 h heart rate recovery (**b**), 24 h rMSSD (**c**) and 24 h SDNN (**d**) across all temperature thresholds.

Our study also found that the influence of training duration on ANS deterioration is augmented at higher core temperatures and the “time to” ANS deterioration is accelerated. The strength of the associations between training duration and ANS recovery and function strengthens with increases in core temperature. Specifically, at the ≥39 °C threshold, we observed a larger magnitude of this association for baseline HR, HR recovery, rMSSD and SDNN (R^2^ = 0.62 vs. 0.26, 0.52 vs. 0.21, 0.58 vs. 0.24 and 0.59 vs. 0.22, respectively). Additionally, we observed a steeper slope between duration and 24 h ANS recovery and function when core temperature increased from the ≥38 °C threshold to ≥39.0 °C (baseline HR: *β* = 0.10 vs. 0.39, HR recovery: *β* = −0.06 vs. −0.26, rMSSD: *β*=−0.11 vs. −0.37 and SDNN: *β* = −0.16 vs. −0.67). This finding suggests that as core temperatures increase beyond 38 °C, ANS deterioration is accelerated. The precipitous deterioration of the ANS following exposure to increasing hyperthermic temperatures is well-established [31,32,33]. Former studies show that sport performance, even at the elite level, significantly declines at very high core temperatures (40.0 °C–41.5 °C) despite adequate heat acclimatization and pre-, during- and post-competition cooling strategies [11,12]. These studies reported varying degrees of dehydration, extreme central and peripheral fatigue, reduced motor function, etc., throughout and immediately post competition. Given the findings of the current study, we speculate that those elite athletes likely experienced some degree of ANS dysfunction 24 h later, but it was not measured. Relatedly, our study also showed that 24 h ANS deterioration may occur following *shorter* bouts of training in the high heat than at sub-hyperthermic core temperatures. For example, in Figure 5, ANS recovery (baseline HR) degradation under the ≥39.0 °C threshold was initiated following only a 10 min bout of training, whereas under the ≥38.0 °C threshold a 60 min exposure was required to elicit ANS degradation. Similarly, deterioration of ANS function (HR recovery, rMSSD and SDNN) followed 10 min and 70 min exposures under the ≥39.0 °C and ≥38.0 °C thresholds, respectively. This indicates that at elevated core temperatures, a longer duration may be required to induce the same level of ANS dysfunction, and vice versa for training under higher core temperatures. This finding further emphasizes the importance of coaches monitoring both core temperature and duration throughout training, as the physiological load amplifies with parallel increases in temperature. The increased physiological load consequent to higher core temperature, however, is deteriorative due to the body working against the heat and not towards improved performance. Core temperature increases rapidly at the onset of exercise due to higher ATP production and hydrolysis. In hot and humid climates, core temperature fails to reach a steady state, continuing to climb until the heat exposure is removed (e.g., cooling intervention) [34]. Without monitoring, an athlete may quickly surpass a core temperature of 38.0 °C and 39.0 °C without acute and observable effects of ANS deterioration.

Interestingly, and rather expectedly, this study found that under core temperature values between 37.0 °C and 37.9 °C, training duration was no longer significantly associated with 24 h ANS recovery and function. This observation was not surprising as resting core temperature fluctuates between 36.1 °C and 37.8 °C. Importantly, as shown in Figure 5, athletes were able to perform exercise longer under the 37.0 °C threshold compared to the 38 °C and 39 °C thresholds before the onset of ANS deterioration. Here, training for a longer duration is physiologically plausible as the ANS can adequately thermoregulate and dissipate metabolic heat at the same rate it is produced [4]. Maintaining this core temperature, however, is only possible when practicing in thermoneutral environments like air-conditioned facilities [35]. Serving as their own control, when practicing indoors at thermoneutral temperatures, the core temperatures of the study sample remained, on average, within normal limits and not surpassing 37.8 °C (data not shown). More importantly, the core temperatures reached during indoor training, regardless of duration, were not associated with ANS deterioration. These findings indicate, at the lower core temperature threshold (within normal limits), that duration of training may not meaningfully impact ANS recovery and function post 24 h. As such, training longer in thermoneutral environments, and including short, intermittent bouts (e.g., 30 to 45 min) outside in environments that may increase core temperature quickly (e.g., hot and humid and/or wearing heat-intolerant clothing), may be the most effective approach for optimizing sports performance. This alternating pattern allows for athletes to “cool off” following exposure to an elevated core temperature and safely continue training for longer durations [36].

There are strengths and weaknesses of the current study that warrant attention. First, this study employed a prospective cohort design in a natural setting, producing higher quality evidence and improved translation of findings. Second, this study was the first, to our knowledge, to observe ANS deterioration 24 h following prolonged exercise at sub-hyperthermic temperature thresholds. This finding highlights the importance of monitoring athletes’ physiological tolerance (e.g., core temperature, ANS function, external load) of exercise training. Third, core temperature was measured using a noninvasive method which was previously validated and strongly correlated with the gold standard measure (Swan–Ganz catheter temperature) [22,23]. There are some limitations to this study. First, the generalizability of our findings is restricted to one D1 college football team in a single geographical location. As such, we cannot assume these findings apply to different sports, levels of competition (e.g., D2, D3) and/or female sports. Second, other factors potentially influencing the magnitude of the observed associations, such as medications (e.g., Adderall), caffeine, sleep and mental stress, were not measured.

## 5. Conclusions

In conclusion, the findings of this study demonstrate that the duration of training negatively influenced 24 h ANS recovery and function when performed at core temperatures ≥38 °C and ≥39.0 °C. Training sessions as short as 5 min at the highest threshold were associated with greater ANS dysfunction. This finding indicates that training while exposed to elevated core temperatures, even in small doses, may elicit a longer-lasting negative impact on ANS recovery function than previously shown. Our observations emphasize the need for coaches to monitor their athletes’ physiological responses to exercise training, especially during summer training camps and preseason training. These training sessions occur during the hottest and most humid months of the year and following a period of detraining in the off-season. Consequently, during this time, athletes may be unaccustomed to training in hot/humid climates, potentially resulting in an accelerated rise in core temperature. Additionally, our findings suggest that for long-duration training sessions, coaches should consider implementing short, intermittent bouts of training if core temperatures are elevated (e.g., 30 to 45 min), and separating them with prolonged “cooling off” periods. We recommend that future studies evaluate the cumulative effect of training at various core temperature thresholds on ANS recovery and function, as collegiate athletes often train 5 to 6 days per week. Consecutive training sessions following exposure to elevated core temperatures may facilitate further ANS deterioration, reducing the ability to thermoregulate and negatively affecting sports performance.

## Figures and Tables

**Table 1:** Duration of acute exercise training sessions, time spent in temperature thresholds and 24 h ANS recovery and function.

	Mean (SD)	Median (Min, Max)
No. of Training Sessions	128	-----
Duration of Sessions (min)	161.1 (40.6)	157.1 (90.1, 339.6)
Duration (min) Under Temperature Thresholds		
≥37 °C	30.4 (35.9)	18.0 (0.0, 121.5)
≥38 °C	19.5 (21.5)	15.0 (0.0, 81.0)
≥39 °C	10.5 (12.9)	2.8 (0.0, 40.5)
24 h ANS Recovery and Function		
Baseline HR (bpm)	61.4 (8.6)	60.1 (44.8, 118.2)
30 s HR Recovery (bpm)	30.6 (6.0)	31.0 (11.2, 49.6
rMSSD (ms)	72.0 (7.0)	72.5 (55.4, 93.2)
SDNN (ms)	106.7 (12.2)	107.6 (81.1, 141.8)

**Table 2:** Adjusted linear regression coefficients for the relationships between time spent in different temperature thresholds and baseline heart rate and heart rate recovery.

	Slope (*β*)	SE	Adjusted R^2^	95% CI	*p*-Value
Baseline HR (bpm)					
Duration Under Temperature Thresholds					
≥37 °C	0.01	0.01	0.03	(−0.02, 0.02)	0.698
≥38 °C	0.10	0.02	0.26	(0.06, 0.14)	*p* < 0.0000
≥39 °C	0.39	0.05	0.62	(0.30, 4.49)	*p* < 0.0000
HR Recovery (bpm)					
Duration Under Temperature Thresholds					
≥37 °C	0.003	0.01	0.02	(−0.01, 0.02)	0.609
≥38 °C	−0.06	0.02	0.21	(−0.10, −0.03)	0.0002
≥39 °C	−0.26	0.04	0.52	(−0.34, −0.18)	*p* < 0.0000

**Table 3:** Adjusted linear regression coefficients for the relationships between time spent under different temperature thresholds and 24 h heart rate variability.

	Slope (*β*)	SE	Adjusted R^2^	95% CI	*p*-Value
rMSSD					
Duration Under Temperature Thresholds					
≥37 °C	−0.01	0.01	0.04	−0.03, 0.02	0.635
≥38 °C	−0.11	0.02	0.24	−0.15, −0.06	*p* < 0.0000
≥39 °C	−0.37	0.05	0.58	−0.47, −0.26	*p* < 0.0000
SDNN					
Duration Under Temperature Thresholds					
≥37 °C	−0.01	0.02	0.027	−0.05, 0.03	0.529
≥38 °C	−0.16	0.04	0.22	−0.24, −0.08	*p* < 0.0000
≥39 °C	−0.67	0.09	0.58	−0.85, −0.49	*p* < 0.0000

## Data Availability

The data can be provided by Tiger Tech Solutions, Inc., pending scientific review and a completed materials transfer agreement. Requests for the data should be submitted to the corresponding author: Samantha M McDonald, smmcdo4@ilstu.edu.

## References

[1] Kellmann M., Bertollo M., Bosquet L., Brink M., Coutts A.J., Duffield R., Erlacher D., Halson S.L., Hecksteden A., Heidari J. (2018). Recovery and Performance in Sport: Consensus Statement. Int. J. Sports Physiol. Perform..

[2] Pincivero D.M., Bompa T.O. (1997). A Physiological Review of American Football. Sports Med..

[3] Fealey R.D., Buijs R.M., Swaab D.F. (2013). Chapter 7—Interoception and Autonomic Nervous System Reflexes Thermoregulation. Handbook of Clinical Neurology.

[4] Cramer M.N., Jay O. (2016). Biophysical Aspects of Human Thermoregulation during Heat Stress. Auton. Neurosci..

[5] Ftaiti F., Grélot L., Coudreuse J.M., Nicol C., Coudreuse J.M. (2001). Combined Effect of Heat Stress, Dehydration and Exercise on Neuromuscular Function in Humans. Eur. J. Appl. Physiol..

[6] Armstrong L.E., Johnson E.C., Casa D.J., Ganio M.S., McDermott B.P., Yamamoto L.M., Lopez R.M., Emmanuel H. (2010). The American Football Uniform: Uncompensable Heat Stress and Hyperthermic Exhaustion. J. Athl. Train..

[7] Krohn A.R., Sikka R., Olson D.E. (2015). Heat Illness in Football: Current Concepts. Curr. Sports Med. Rep..

[8] Yeargin S.W., Kerr Z.Y., Casa D.J., Djoko A., Hayden R., Parsons J.T., Dompier T.P. (2016). Epidemiology of Exertional Heat Illnesses in Youth, High School, and College Football. Med. Sci. Sports Exerc..

[9] Kay D., Marino F.E. (2000). Fluid Ingestion and Exercise Hyperthermia: Implications for Performance, Thermoregulation, Metabolism and the Development of Fatigue. J. Sports Sci..

[10] Nybo L., Nielsen B. (2001). Hyperthermia and Central Fatigue during Prolonged Exercise in Humans. J. Appl. Physiol..

[11] Beal H., Corbett J., Davis D., Barwood M.J. (2022). Marathon Performance and Pacing in the Doha 2019 Women’s IAAF World Championships: Extreme Heat, Suboptimal Pacing, and High Failure Rates. Int. J. Sports Physiol. Perform..

[12] Racinais S., Nichols D., Travers G., Moussay S., Belfekih T., Farooq A., Schumacher Y.O., Périard J.D. (2020). Health Status, Heat Preparation Strategies and Medical Events among Elite Cyclists Who Competed in the Heat at the 2016 UCI Road World Cycling Championships in Qatar. Br. J. Sports Med..

[13] Grundstein A.J., Hosokawa Y., Casa D.J. (2018). Fatal Exertional Heat Stroke and American Football Players: The Need for Regional Heat-Safety Guidelines. J. Athl. Train..

[14] Eichner E.R. (2021). The Heat Is On: Exertional Heatstroke in Football. Curr. Sports Med. Rep..

[15] Eichner E.R. (2019). Fatal Exertional Heat Stroke in Football: The Coaches Are the Culprits. Curr. Sports Med. Rep..

[16] Davis J.K., Baker L.B., Barnes K., Ungaro C., Stofan J. (2016). Thermoregulation, Fluid Balance, and Sweat Losses in American Football Players. Sports Med..

[17] Launstein E.D., Miller K.C., O’Connor P., Adams W.M., Abrego M.L. (2021). American Football Uniforms Elicit Thermoregulatory Failure during a Heat Tolerance Test. Temperature.

[18] Peck J., Wishon M.J., Wittels H., Lee S.J., Hendricks S., Davila H., Wittels S.H. (2021). Single Limb Electrocardiogram Using Vector Mapping: Evaluation and Validation of a Novel Medical Device. J. Electrocardiol..

[19] Wittels S.H., Renaghan E., Wishon M.J., Wittels H.L., Chong S., Wittels E.D., Hendricks S., Hecocks D., Bellamy K., Girardi J. (2023). Recovery of the Autonomic Nervous System Following Football Training among Division I Collegiate Football Athletes: The Influence of Intensity and Time. Heliyon.

[20] Renaghan E., Wittels H.L., Feigenbaum L.A., Wishon M.J., Chong S., Wittels E.D., Hendricks S., Hecocks D., Bellamy K., Girardi J. (2023). Exercise Cardiac Load and Autonomic Nervous System Recovery during In-Season Training: The Impact on Speed Deterioration in American Football Athletes. J. Funct. Morphol. Kinesiol..

[21] Wittels S.H., Renaghan E., Wishon M.J., Wittels H.L., Chong S., Wittels E.D., Hendricks S., Hecocks D., Bellamy K., Girardi J. (2023). A Novel Metric “Exercise Cardiac Load” Proposed to Track and Predict the Deterioration of the Autonomic Nervous System in Division I Football Athletes. J. Funct. Morphol. Kinesiol..

[22] Gómez-Romero F.J., Fernández-Prada M., Fernández-Suárez F.E., Gutiérrez-González C., Estrada-Martínez M., Cachero-Martínez D., Suárez-Fernández S., García-González N., Picatto-Hernández M.D., Martínez-Ortega C. (2019). Intra-Operative Temperature Monitoring with Two Non-Invasive Devices (3M Spoton^®^ and Dräger Tcore^®^) in Comparison with the Swan-Ganz Catheter. Cirugía Cardiovasc..

[23] Sessler D.I. (2021). Perioperative Temperature Monitoring. Anesthesiology.

[24] Speed C., Arneil T., Harle R., Wilson A., Karthikesalingam A., McConnell M., Phillips J. (2023). Measure by Measure: Resting Heart Rate across the 24-Hour Cycle. PLoS Digit. Health.

[25] Nandi P.S., Spodick D.H. (1977). Recovery from Exercise at Varying Work Loads. Time Course of Responses of Heart Rate and Systolic Intervals. Br. Heart J..

[26] Task Force of the European Society of Cardiology, North American Society of Pacing and Electrophysiology (1996). Heart rate variability. Standards of measurement, physiological interpretation, and clinical use. Circulation.

[27] Reilly T., Ekblom B. (2005). The Use of Recovery Methods Post-exercise. J. Sports Sci..

[28] Altarriba-Bartes A., Pena J., Vicens-Bordas J., Mila-Villaroel R., Calleja-Gonzalez J. (2020). Post-Competition Recovery Strategies in Elite Male Soccer Players. Effects on Performance: A Systematic Review and Meta-Analysis. PLoS ONE.

[29] Burke S., Hanani M. (2012). The Actions of Hyperthermia on the Autonomic Nervous System: Central and Peripheral Mechanisms and Clinical Implications. Auton. Neurosci..

[30] Stamatis A., Magnusen M. (2021). Nontraumatic Injuries in the NCAA: Collegiate Football Strength Coaches Should Exercise Caution This Off-Season. Int. J. Exerc. Sci..

[31] Mtibaa K., Thomson A., Nichols D., Hautier C., Racinais S. (2018). Hyperthermia-Induced Neural Alterations Impair Proprioception and Balance. Med. Sci. Sports Exerc..

[32] Nybo L., Rasmussen P., Sawka M.N. (2014). Performance in the Heat-Physiological Factors of Importance for Hyperthermia-Induced Fatigue. Compr. Physiol..

[33] Periard J.D., Racinais S. (2016). Performance and Pacing during Cycle Exercise in Hyperthermic and Hypoxic Conditions. Med. Sci. Sports Exerc..

[34] Nadel E.R., Cafarelli E., Roberts M.F., Wenger C.B. (1979). Circulatory Regulation during Exercise in Different Ambient Temperatures. J. Appl. Physiol..

[35] Fernandez A., Wimer G.S., Culver M.N., Flatt A.A., Grosicki G.J. (2023). Fan Cooling Improves Submaximal Exercise Capacity in an Indoor Thermoneutral Environment. Res. Quart. Exerc. Sport..

[36] Bradley L.J., Miller K.C., Wiese B.W., Novak J.R. (2019). Precooling’s Effect on American Football Skills. J. Strength. Cond. Res..

